# Health-related quality of life and utility in head and neck cancer survivors

**DOI:** 10.1186/s12885-019-5614-4

**Published:** 2019-05-07

**Authors:** Li-Jen Liao, Wan-Lun Hsu, Wu-Chia Lo, Po-Wen Cheng, Pei-Wei Shueng, Chen-Hsi Hsieh

**Affiliations:** 1 Department of Otolaryngology, Head and Neck Surgery, Far Eastern Memorial Hospital, New Taipei City, Taiwan; 2 Department of Electrical Engineering, Yuan Ze University, Taoyuan, Taiwan; 3 Genomics Research Center, Academia Sinica, Taipei, Taiwan; 4 Division of Radiation Oncology, Department of Radiology, Far Eastern Memorial Hospital, No. 21, Sec. 2, Nanya S. Rd., Banciao Dist., New Taipei City, 220 Taiwan; 5 Faculty of Medicine, School of Medicine, National Yang-Ming University, Taipei, Taiwan; 6 Institute of Traditional Medicine, School of Medicine, National Yang-Ming University, Taipei, Taiwan

**Keywords:** Disability, Head and neck cancer, Quality of life, Radiotherapy, Utility

## Abstract

**Background:**

This study seeks to assess quality of life (QOL) and utility scores of head and neck cancer survivors.

**Methods:**

We compared QOL as indicated by EORTC QLQ-C30, QLQ-H&N35, utility scores by time trade off (TTO) with previous published reference values and tested series characteristics related to global QOL and utility.

**Results:**

A total of 127 patients were recruited. Of the patients, 102 (80%) completed the utility assessment. Cancer survivors had lower scores compared with norm values. Patients without a spouse had a lower utility than those with a spouse. Patients with a low annual family income also had lower global QOL and utility scores (*p* < 0.05). Other factors were not significantly related to QOL and utility scores.

**Conclusion:**

Disease and treatment of head and neck cancer lead to disability and poor health-related QOL and utility. Economic status may contribute to health-related QOL and utility, while marital status is related to utility for head and neck cancer patients.

## Background

The incidence of head and neck cancer is increasing, and survival rates are improving, with the overall 5-year relative survival rate increasing from 54.7% in 1992–1996 to 65.9% in 2002–2006 [1]. Even if cancer is eliminated, the treatment of head and neck cancer (HNC) survivors may be complicated with disability [2, 3]. In addition to survival, quality of life (QOL) is also important for head and neck cancer survivors. Hence, outcome evaluations of cancer treatment should include not only survival but also disability [4]. Patient-reported health-related QOL and utility scores are important measurements of these disabilities, which indicate the overall well-being of patients [5].

Today, QOL is increasingly considered among the study endpoints. Patient choices, clinical decision-making and resource allocation all take survival and QOL into consideration simultaneously. Several decision-analysis studies based on utility theory have been reported [6–8]. Due to significant functional limitations related to the disease and treatment, the QOL in long-term head and neck cancer survivors is reportedly worse compared with the general population [9, 10]. However, most studies focus on the assessment of QOL; only a few studies have focused on utility assessment in head and neck cancer survivors. The utility in laryngeal cancer is significantly correlated to performance status and is perceived differently by patients [11]. The characteristics related to the health-related QOL and utility of head and neck cancer survivors have never been assessed in detail.

Divorced, never-married and widowed men exhibit higher mortality rates relative to married men, suggesting that a closer supportive relationship may have an important impact on survival and QOL [12, 13]. Socioeconomic status also has an important impact on the survival time from diagnosis [14]. Social network is positively correlated with social support and positively regressed on income [15]. Herein, we also found a significant relationship for economic status with global QOL and utility. As mentioned previously, social welfare systems may need to offer more support to HNC patients with a lower annual family income to increase the QOL and utility.

Several factors may affect the QOL and utility of head and neck cancer survivors, such as disease severity, cancer types, marry status and socioeconomic status. The current study assesses the utility values with time trade off (TTO) of head and neck cancer survivors by the European Organization for Research and Treatment of Cancer Quality of Life Questionnaire-Core 30 (EORTC QLQ-C30) version 3 and QLQ-H&N35 scores as well as marital and socioeconomic status related to these scores at a single medical center.

## Materials and methods

### Study design and patients

This was a prospective study. The study sample included 127 patients with advanced HNC treated between February 2016 and August 2016 in Far Eastern Memorial Hospital in Taiwan. The study protocol was approved by the institutional review board (FEMH 104078-E). The inclusion criterion include pathology-diagnosed head and neck cancers; patients who completed definitive therapy and were disease free for more than 6 months after the definitive treatment were invited to join our study. Radiotherapy techniques included three-dimensional conformal radiation therapy (3DCRT), intensity modulated radiation therapy (IMRT), volumetric modulated arc therapy (VMAT) and helical tomotherapy (HT). Patients who had not undergone definitive therapy, who were post-therapy less than 6 months, and who had local or regional disease were excluded. Participants were invited to an outpatient clinic. All enrolled participants signed an inform consent form that was approved by the Institutional Review Board of the Far Eastern Memorial Hospital (FEMH 104078-E).

### QOL questionnaires

A trained research assistant assessed demographic data and QOL using the European Organization for Research and Treatment of Cancer Quality of Life Questionnaire-Core 30 (EORTC QLQ-C30) version 3 and QLQ-H&N35. The validated Taiwan Chinese version was employed [16, 17]. Patients completed the EORTC QLQ-C30 (version 3.0) and QLQ-H&N35 questionnaires before the start of treatment and at a regular follow-up visit after completing active treatment. The scores of the QLQ-C30 and QLQ-H&N35 items were linearly transformed to 0–100 scales. For functioning scales and global QOL scales, higher scores correspond to better levels of functioning. For symptom scales, higher scores represent higher levels of symptoms or problems [18]. All patients were included in a prospective follow-up program, and QOL was assessed 1.5 months after completion of radiotherapy under disease-free status. We used recently published Swedish population-based norm reference values and head and neck cancer scores [3] to compare our results.

#### EORTC QLQ-C30 version 3

The QLQ-C30 is composed of both multi-item scales and single-item measures, including five functional scales, three symptom scales, a global health status/QOL scale, and six single items. All the scales range from 0 to 100. A high score on the functional scales represents a high level of functioning, and a high score on the symptom scales represents a high level of symptomatology. A high score on the global QOL represents a high general quality of life. The manual contains scoring procedures for QLQ-C30 version 3.0 and QLQ-C30 version 3.0, which are used in the current studies. All scales were scored in accordance with the EORTC scoring manual [19, 20].

#### EORTC QLQ-H&N35

The QLQ-H&N35 is a module used for assessing the QOL specifically in head-and-neck cancer patients. QLQ-H&N35 incorporates seven multiple-item scales that assess the symptoms of pain, swallowing ability, senses (taste/smell), speech, social eating, social contact, and sexuality. Six single-item scales are also included that survey the presence of symptomatic problems associated with the teeth, mouth opening, dry mouth (xerostomia), sticky saliva, coughing, and malaise. A high score on the symptom scales represents a high level of symptomatology.

#### Utility instrument

TTO has previously been used to assess laryngeal utility in several studies [11]. We used TTO but not EQ-5D as our measurement technique for head and neck cancer survivors given that TTO is a “choice task” not a “rating task” that easily involves some scaling bias [21]. Time trade off is recommended when performing cost-utility analysis using quality-adjusted life years as an outcome. The patients were first asked to imagine how many years they had left to live (X). Then, they could choose to give up some life years (Y) to live for a shorter period in perfect health. The utility would then be (X-Y)/X, according to the TTO method. The values are anchored at 1 (full health) and 0 (dead); the higher values mean higher health utility. Utility and QOL were assessed simultaneously.

### Statistical analysis

For descriptive purposes, the mean and standard deviations (SDs) of the variables were used for continuous parameters. The nonparametric model was used to compare continuous variables. Nonparametric Mann-Whitney tests were employed for differences among two groups. Kruskal-Wallis tests and sequential post hoc tests were used for analyses between multiple groups. Category parameters were expressed as an absolute number and percentage (%) and were compared by the χ^2^ test or Fisher’s exact test among groups, when appropriate. We used two-sample t-tests with summary statistics for means, standard deviations, and sample sizes to compare study groups with previous published reference norms and data for head and neck cancer survivors [3]. The correlation between utility and global health status was assessed with the Spearman rank correlation coefficient. Statistical analysis was performed using STATA software, version 12.0 (Stata Statistical Software: Release 12; Stata Corp LP, College Station, TX).

## Results

From Feb. 2016 to Aug. 2016, 139 patients who met the inclusion were approached, and 127 (91.4%) patients were enrolled after informed consent. The mean follow-up duration after definite treatment is 40 months. In total, 127 patients were recruited, including 51 oral cancer patients, 24 nasopharyngeal carcinoma patients, 17 thyroid cancer patients, 15 oropharyngeal cancer patients, 10 hypopharyngeal cancer patients, and 10 laryngeal cancer patients. All patients completed QOL questionnaires, and 102 patients completed the utility assessment employing the TTO method. Details and treatment strategies are summarized in Table 1.

**Table 1:** Patient characteristics and medically related variables for head and neck cancer survivors

Characteristic	Total	%
Age, mean (SD), years	56.7 (10.1)	
Gender		
Female	20	16%
Male	107	84%
Tumor site		
Oral cancer	51	40%
Nasopharyngeal cancer	24	19%
Thyroid cancer	17	12%
Oropharyngeal cancer	15	8%
Laryngeal cancer	10	8%
Hypopharyngeal cancer	10	13%
Education (years)		
Less than 6 years	21	17%
6–12 years	97	77%
More than 12 years	9	7%
Occupational status		
Employed	62	49%
Homemaking	5	4%
None	60	47%
Marital status		
With a spouse	95	75%
Without a spouse	32	25%
Annual family income		
> 1,000,000 NTD	88	69%
500,000~1,000,000 NTD	32	25%
< 500,000 NTD	7	6%
Habits related to cancer		
Tobacco use	81	64%
Betel nut use	58	46%
Alcohol use	63	50%
Comorbidity (YES)	53	42%
AJCC (7th edition) Stage		
I	36	28%
II	15	12%
III	23	18%
IV	50	39%
Treatment		
Chemotherapy		
Yes	80	66%
No	41	34%
Radiation therapy		
Yes	96	79%
No	26	21%
Surgery		
Yes	98	85%
No	18	15%
F/U time, mean (SD), years	3.3(0.2)	

I US$ ~33NTD; *AJCC* American joint committee on cancer

### EORTC QLQ-C30

Details of the QOL assessment using the EORTC QLQ-C30 are summarized in Table 2, and recently published reference data [3] are presented for comparison. The global health statuses (QOL, mean and SD) were 74.7 (19.9) for oral cancer, 66.0 (19.8) for NPC, 66.7 (29.1) for oropharyngeal cancer, 71.7 (19.7) for hypopharyngeal cancer, 69.2 (30.4) for laryngeal cancer, and 67.6 (22.4) for thyroid cancer (*p* = 0.5, Kruskal-Wallis test). Compared with published references of normal values, our study HNC patients had lower global health status [70.9 (22.1) versus 76.6 (22.0), *p* < 0.01]. However, no significant difference was noted compared with published HNC survivors [70.9 (22.1) versus 73.2 (21.3), *p* = 0.4].

**Table 2:** Results from the EORTC QLQ-C30 version 3.0 and utility assessment of head and neck cancer survivors. A comparison of head and neck cancer patients with a report from the Swedish population [3]

	Oral cancer (*n* = 51)	Nasopharyngeal cancer (*n* = 24)	Oropharyngeal cancer (*n* = 15)	Hypopharyngeal cancer (*n* = 10)	Laryngeal cancer (*n* = 10)	Thyroid cancer (*n* = 17)	Total (*n* = 127)	Published reference norm values (*n* = 562)	*p*1	Published Head and neck cancer survivors	*p*2
										(*n* = 133)	
	Mean (SD)	Mean (SD)	Mean (SD)	Mean (SD)	Mean (SD)	Mean (SD)	Mean (SD)	Mean (SD)		Mean (SD)	
Functional scales											
Physical functioning	89.80 (20.28)	93.05 (8.67)	90.67 (12.03)	94(8.58)	75.33 (27.41)	96.86 (5.33)	90.66 (16.78)	88.9 (18.1)	0.28	85.6 (21.2)	0.03
Role functioning	95.42 (20.02)	95.83 (14.95)	95.56 (17.21)	100	88.33 (22.29)	100	95.93 (16.63)	86.5 (24.8)	< 0.01	83.3 (30.1)	< 0.01
Emotional functioning	88.73 (15.08)	86.81 (19.18)	92.78 (14.04)	94.17 (11.15)	93.33 (10.97)	81.86 (18.22)	88.71 (15.87)	84.6 (20.1)	0.01	85.0 (18.7)	0.09
Cognitive functioning	85.29 (17.53)	81.94 (13.83)	84.44 (18.33)	95 (11.25)	83.33 (15.71)	78.43 (18.41)	84.25 (16.7)	89.5 (20.6)	< 0.01	86.0 (22.2)	0.4
Social functioning	92.16 (19.25)	95.83 (8.86)	97.78 (8.61)	96.67 (7.03)	93.33 (19.56)	92.16 (10.40)	93.96 (14.80)	86.9 (18.1)	< 0.01	87.0 (16.5)	< 0.01
Symptom scales/items											
Fatigue	16.56 (24.68)	14.81 (19.29)	14.07 (27.69)	10 (15.23)	18.89 (20.98)	17.65 (20.05)	15.75 (22.33)	19.7 (21.4)	0.07	22.8 (22.9)	0.01
Nausea and vomiting	2.29 (7.47)	2.78 (8.03)	0	5 (11.25)	3.33 (10.54)	1.96 (8.09)	2.36 (7.78)	3.3 (11.0)	0.28	3.1 (9.0)	0.5
Pain	17.97 (25.9)	10.42 (13.74)	10 (16.43)	16.67 (22.22)	26.67 (33.52)	8.82 (8.58)	14.96 (21.91)	17.7 (24.6)	0.22	14.9 (20.7)	0.97
Dyspnea	6.54 (20.02)	2.78 (9.41)	8.89 (19.79)	6.67 (14.06)	13.33 (23.31)	1.96 (8.09)	6.04 (17.02)	16.5 (24.1)	< 0.01	17.8 (23.9)	< 0.01
Insomnia	24.2 (36.6)	9.7 (18.3)	8.9 (26.6)	23.3 (31.6)	20 (28.1)	25.5 (27.7)	19.4 (30.7)	16.5 (24.9)	0.32	18.8 (26.4)	0.87
Appetite loss	8.50 (21.95)	11.11 (25.38)	15.56 (30.52)	16.67 (17.57)	16.67 (28.33)	7.84 (14.57)	11.02 (23.02)	3.4 (12.7)	< 0.01	11.7 (25.1)	0.81
Constipation	11.76 (19.80)	9.72 (20.80)	11.11 (27.22)	6.67 (14.05)	6.67 (14.05)	17.65 (20.81)	11.28 (20.24)	5.1 (15.9)	< 0.01	3.8 (11.4)	< 0.01
Diarrhea	4.58 (16.35)	4.17 (11.26)	0	3.33 (10.54)	3.33 (10.54)	17.65 (29.15)	5.51 (16.67)	5.0 (16.0)	0.75	10.8 (21.9)	0.03
Financial difficulties	7.19 (16.75)	5.56 (16.05)	11.11 (27.22)	10 (22.50)	16.67 (23.57)	5.88 (13.10)	8.14 (18.65)	6.6 (19.7)	0.41	11.6 (25.2)	0.2
Global health status/QOL											
Global health status/QOL	74.67 (19.86)	65.97 (19.80)	70 (28.49)	71.67 (19.72)	69.17 (30.44)	67.65 (22.42)	70.87 (22.09)	76.5 (22.0)	< 0.01	73.2 (21.3)	0.4
Utility (*N* = 102)	0.71 (0.17)	0.73 (0.20)	0.76 (0.14)	0.72 (0.24)	0.70 (0.20)	0.70 (0.21)	0.7 (0.2)	NA	NA	NA	NA

*p1 P*-value between the study population and the reference norm, *p2 P*-value between the study population and previously published head and neck cancer survivors

### Correlation of global QOL and utility

The mean global health status score was 70.9 (22.1) for recruited patients, 74.7 (19.9) for oral cancer, 66.0 (19.8) for NPC, 66.7 (29.1) for oropharyngeal cancer, 71.7 (19.7) for hypopharyngeal cancer, 69.2 (30.4) for laryngeal cancer, and 67.6 (24.4) for thyroid cancer (p = 0.5, Kruskal-Wallis test). The overall mean utility index was 0.7 (0.2). The mean utility index was 0.71 (0.17) for oral cancer, 0.73 (0.20) for NPC, 0.76 (0.14) for oropharyngeal cancer, 0.72 (0.24) for hypopharyngeal cancer, 0.70 (0.20) for laryngeal cancer, and 0.70 (0.21) for thyroid cancer (*p* = 0.98, Kruskal-Wallis test). Spearman’s correlation was performed to assess the relationship between utility and global health status using 102 participants. A positive correlation was noted between utility and global health status, which had a low level of significance (*r*_*s*_ = 0.24, *p* = 0.02).

### EORTC QLQ- HN35

The results of EORTC QLQ- HN35 are summarized in Table 3. Thyroid cancer caused fewer sense problems with a score of 2 (5.5). Dry mouth yielded a score of 2 (8.1), which is lower than other cancers, and oropharyngeal cancer caused more cough problems, yielding a score of 53.3 (35.2) (*p*-value < 0.05 for the Kruskal-Wallis and post hoc tests). Otherwise, there was no significant difference among the different cancers. Compared with published references of normal values (Table 3), our study HNC patients had higher symptoms scales [*p* < 0.01, except trouble with social contact]. Compared with published references of HNC patients, our study group had lower symptom scales in pain [8.1 (16.5) versus 14.5 (19.8), *p* < 0.01], sense problems [12.1 (21.87) versus 20.5 (27.4), *p* < 0.01], less sexuality [13.7 (37.5) versus 28.8 (36.3), *p* < 0.01], mouth opening [27.8 (33.8) versus 17.6 (29.3), *p* < 0.01], dry mouth [21.3 (33.5) versus 47.3 (36.3), *p* < 0.01], coughing [26.0 (33.6) versus 17.3 (24.6), *p* = 0.02] and malaise [18.6 (29.0) versus 10.8 (20.0), *p* = 0.01]. Our study patients had lower symptom scales in sticky saliva [35.2 (34.7) versus 18.6 (28.4), *p* < 0.01]. There were no differences in symptoms scales in swallowing, speech problems, trouble with social eating, and trouble with social contact between these two groups of HNC survivors (all *p* > 0.05).

**Table 3:** Results of the EORTC QLQ-HN35 for head and neck cancer survivors. A comparison of the head and neck cancer patients with a report from the Swedish population [3]

Symptom scales/items	Oral cancer (*n* = 51)	Nasopharyngeal cancer (*n* = 24)	Oropharyngeal cancer (*n* = 15)	Hypopharyngeal cancer (*n* = 10)	Laryngeal cancer (*n* = 10)	Thyroid cancer (*n* = 17)	Total (*n* = 127)	Published Reference norm values (*n* = 562)	*p*1	Published Head and neck cancer survivors (*n* = 133)	*p*2
	Mean (SD)	Mean (SD)	Mean (SD)	Mean (SD)	Mean (SD)	Mean (SD)	Mean (SD)	Mean (SD)		Mean (SD)	
Pain	10.13 (18.66)	6.60 (12.03)	11.67 (26.50)	4.17 (8.10)	7.5 (10.72)	3.92 (8.90)	8.14 (16.52)	3.0 (9.4)	< 0.01	14.5 (19.8)	< 0.01
Swallowing	9.80 (17.46)	9.72 (25.38)	18.33 (24.44)	8.33 (15.71)	14.17 (22.59)	6.37 (11.98)	10.56 (19.71)	2.0 (7.2)	< 0.01	10.9 (19.6)	0.9
Sense problems	7.19 (18.33)	18.06 (25.97)	22.22 (24.13)	16.67 (19.25)	21.67 (32.44)	1.96 (5.54)	12.21 (21.87)	5.8 (16.1)	< 0.01	20.5 (27.4)	< 0.01
Speech problems	12.20 (19.41)	8.33 (15.46)	20 (25.27)	1.11 (19.91)	15.56 (28.30)	8.50 (8.36)	11.29 (19.50)	6.1 (13.2)	< 0.01	12.1 (19.1)	0.73
Trouble with social eating	17.81 (27.64)	12.15 (21.14)	27.22 (30.78)	12.5 (20.88)	10.83 (26.07)	2.94 (10.18)	14.90 (25.04)	2.7 (10.0)	< 0.01	11.7 (22.6)	0.28
Trouble with social contact	9.15 (18.81)	4.72 (11.37)	2.67 (6.07)	2.67 (4.66)	10.67 (24.78)	3.92 (5.80)	6.46 (15.05)	3.8 (10.6)	0.05	7.6 (14.7)	0.55
Less sexuality	12.75 (36.15)	11.11 (37.64)	31.11 (47.50)	6.67 (25.09)	10 (30.63)	10.78 (42.06)	13.65 (37.53)	26.1 (33.6)	< 0.01	28.8 (36.3)	< 0.01
Dental problems	32.68 (36.20)	27.78 (28.94)	20 (35.19)	43.33 (44.58)	23.33 (31.62)	13.73 (20.62)	27.82 (33.80)	10.1 (21.4)	< 0.01	21.4 (32.3)	0.12
Mouth opening	32.68 (36.20)	27.78 (28.94)	20 (35.19)	43.33 (44.58)	23.33 (31.62)	13.73 (20.62)	27.82 (33.80)	1.8 (10.8)	< 0.01	17.6 (29.3)	< 0.01
Dry mouth	30.07 (39.58)	16.67 (26.01)	31.11 (36.66)	10 (22.50)	16.67 (32.39)	1.96 (8.09)	21.26 (33.51)	12.3 (22.3)	< 0.01	47.3 (36.3)	< 0.01
Sticky saliva	37.25 (38.09)	33.33 (31.08)	42.22 (36.66)	36.67 (39.91)	33.33 (31.43)	25.49 (27.71)	35.17 (34.71)	6.9 (17.5)	< 0.01	18.6 (28.4)	< 0.01
Coughing	20.92 (31.24)	31.94 (34.72)	53.33 (35.18)	23.33 (35.31)	20 (32.20)	13.73 (26.51)	25.98 (33.57)	16.8 (24.4)	< 0.01	17.3 (24.6)	0.02
Malaise	17.65 (27.77)	18.06 (29.45)	24.44 (40.76)	20 (32.20)	16.67 (32.39)	17.65 (17.15)	18.64 (28.99)	11.3 (21.5)	< 0.01	10.8 (20.0)	0.01

*p1 P*-value between the study population and the reference norm, *p2 P*-value between the study population and previously published head and neck cancer survivors

### Factors related to global QOL and utility

**Fig. 1:**
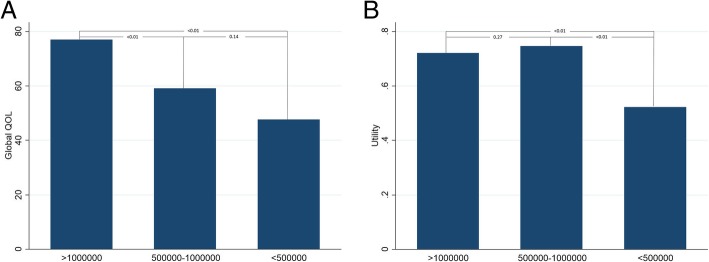
Bar plots show the mean global health (QOL) utility. Post hoc comparisons of global QOL (**a**) and utility (**b**) among different groups of annual family income (classified into > 1,000,000, between 1,000,000 and 500,000 and < 500,000 NTD). The results reveal that a lower annual family income yielded a lower global QOL and utility compared with the other groups

We assessed various factors related to global health status (QOL) and utility, and the results are listed in Table 4. Patients without a spouse had a lower utility compared with patients who had a spouse (p = 0.02). Patients with a lower annual family income also had a lower global QOL and utility. The other factors were not significantly related. Further post hoc test results are shown in Fig. 1. Patients with a lower annual family income (less than 500,000 NTD) had a lower global QOL and utility compared with other groups (*p* < 0.05). Age, gender, tumor site, disease stage, radiation modalities and follow-up time less than or greater than 1 year were not significantly associated with the global health status and utility (*p* > 0.05).

**Table 4:** Comparison of global QOL, utility and various variables in head and neck cancer survivors

Characteristic	Global QOL Mean (SD)	*P*-value	Utility Mean (SD)	*P*-value
Age				
< 65 y/o	70.1 (21.8)	0.46*	0.7 (0.18)	0.99*
≥ 65 y/o	73.7 (23.4)		0.72 (0.19)	
Gender				
Female	66.3 (27.4)	0.31*	0.75 (0.17)	0.40*
Male	71.7 (21.0)		0.71 (0.19)	
Tumor site				
Oral	74.7 (19.9)	0.68†	0.71 (0.17)	0.98†
Nasopharyngeal	66.0 (19.8)		0.73 (0.20)	
Thyroid	67.6 (22.4)		0.70 (0.21)	
Oropharyngeal	66.7 (29.1)		0.76 (0.14)	
Laryngeal	69.2 (30.4)		0.70 (0.20)	
Hypopharyngeal	71.7 (19.7)		0.72 (0.24)	
Education (years)				
Less than 6 years	76.6 (20.9)	0.37†	0.76 (0.18)	0.59†
6–12 years	70.1 (22.0)		0.71 (0.19)	
More than 12 years	65.7 (25.5)		0.67 (0.18)	
Occupational status				
Employed	71.8 (20.0)	0.85†	0.75 (0.17)	0.14†
Homemaking	66.7 (23.6)		0.70 (0.13)	
No	70.3 (24.3)		0.68 (0.20)	
Marital status				
With a spouse	72.1 (22.4)	0.28*	0.74 (0.17)	0.02*
Without a spouse	67.2 (21.2)		0.64 (0.21)	
Annual family income				
> 1,000,000 NTD	77.0 (19.4)	< 0.01†	0.72 (0.18)	0.02†
500,000~1,000,000 NTD	59.1 (21.4)		0.75 (0.20)	
< 500,000 NTD	47.6 (22.9)		0.52 (0.12)	
Habits related to cancer				
Tobacco use (+)	72.0 (20.5)	0.44*	0.72 (0.18)	0.85*
Tobacco use (−)				
Betel nut use (+)	69.8 (20.5)	0.63*	0.71 (0.18)	0.75*
Betel nut use (−)				
Alcohol use (+)	71.4 (22.4)	0.78*	0.74 (0.18)	0.24*
Alcohol use (+)				
AJCC(7th edition) Stage				
I	75 (20.7)	0.59†	0.72 (0.19)	0.98†
II	72.2 (23.3)		0.73 (0.11)	
III	70.7 (19.3)		0.72 (0.20)	
IV	68.3 (24.1)		0.71 (0.18)	
Treatment				
Chemotherapy				
Yes	70.2 (22.3)	0.49*	0.73 (0.18)	0.90*
No	73.2 (21.9)		0.72 (0.17)	
Radiation therapy				
Yes	69.9 (21.6)	0.24*	0.73 (0.18)	0.50*
No	75.6 (23.4)		0.70 (0.18)	
Radiotherapy course	73.5 (21.6)	0.03*	0.70 (0.20)	0.40
Received 1st course	71.2 (21.3)		0.74 (0.17)	
Received 2nd course	56.6 (23.8)		0.67 (0.22)	
Radiotherapy modalities		0.38		0.95
3D CRT+ IMRT	61.7 (21.7)		0.78 (0.14)	
IMRT	63.0 (24.8)		0.73 (0.20)	
VMAT	75 (20.4)		0.72 (0.21)	
HT	71.9 (21.7)		0.72 (0.17)	
Surgery				
Yes	71.4 (22.4)	0.40*	0.71 (0.18)	0.26*
No	66.7 (20.6)		0.77 (0.18)	
F/U time				
< 1 year	67.4 (18.1)	0.41	0.66 (0.19)	0.12
≧ 1 year	71.6 (22.9)		0.73 (0.18)	

I US$ ~33NTD; *AJCC* American joint committee on cancer

*Abbreviations*: *3DCRT* Three-dimensional conformal radiation therapy, *HT* Helical tomotherapy, *IMRT* Intensity-modulated radiation therapy, *VMAT* Volumetric-modulated arc therapy

*Mann-Whitney test

†Kruskal-Wallis test

## Discussion

Several studies have shown low QOL and utility for head and neck cancer survivors [3, 9]. Our study is comparable and further tested factors related to QOL and utility. In our investigation, age, gender, tumor site, disease stage and treatment modalities were not significantly associated with global health status and utility. We found that two factors, marital status and low annual family income, had significant impacts on global QOL and utility.

In this study, we used the TTO method to measure the utility. These values are anchored at 1 (full health and absent any symptom or disease) and 0 (defined as dead); the higher values mean higher health utility. In heart disease, a utility of 0.7 is reported for angina [22]. For head and neck cancer patients alive after selective neck dissection, the utility was 0.97 [23]. Hammerlid et al. reported that the largest health-related quality of life (HRQOL) changes for HNC patients are observed within the first year after diagnosis with a significant deterioration immediately after completing treatment [24]. Noel et al. found that the utility measured by the indirect health state utility value (HSUV) measure for HNC survivors, EuroQoL instrument (EQ-5D) and HU Index Mark 3 (HUI3) ranged from 0.82 to 0.75 [25]. Morillo E et al. reported that the median EQ-5D utility value ranged from 0.7 at baseline for patients with confirmed metastatic and/or recurrent HNC [26]. In the current study, utility had a positive correlation with global QOL and ranged from 0.70 to 0.76 for HNC survivors. The global QOL scales were also lower for our head and neck cancer survivors. Therefore, the deteriorated ability of head and neck cancer survivors can cause a lower HRQOL and further impair patient-perceived utility.

Loimu et al. noted that laryngeal carcinoma patients had a higher HRQoL than patients with pharyngeal carcinoma [27]. However, only 28% of the patients had stage I HNC, and only 8% of the HNC patients had laryngeal cancer in our study. Moreover, Govers et al. observed that invasive procedures result in a lower health utility for oral cavity cancer, even in patients with cT1–2 oral cavity squamous cell carcinoma [28]. Approximately 80% of our patients received invasive treatment, such as surgery, radiotherapy and chemotherapy, which may be partly responsible for the lower health utility. For postoperative patients after total laryngectomy, the functional and QOL outcomes range from intermediate to high categories [29]. A positive correlation was noted between utility and global health status, which had a low level of significance (*r*_*s*_ = .24, *p* = 0.02). These published observations collectively suggest that the participants’ characteristics potentially affected the utility and QOL.

In the current study, significant improvements in treatment and survival for head and neck cancer have been demonstrated for marital status with higher utility for those metrics that did not include spouse status; patients without a spouse had a lower utility compared with patients who had a spouse (*p* < 0.05). Zheng et al. showed that divorced, never-married and widowed men show higher mortality rates relative to married men [12]. Additionally, married patients were less likely to present with metastatic disease, more likely to receive definitive therapy, and less likely to die as a result of their cancer after adjusting for demographics, stage, and treatment compared with unmarried patients [30]. The possible reasons include caregiving in times of illness or poor health, reduction of stress and stress-related illness and general and familial social integration [31]. Marriage also may encourage healthy behaviors and discourage risky or unhealthy behaviors [12]. Umberson found that marriage and parenthood reduce the incidence of health-threatening behaviors, such as problem drinking, substance abuse, and other forms of risk taking [13]. The results from the abovementioned studies and the present investigation indicate that a closer supportive relationship has an important impact on cancer detection, treatment, survival and HRQOL.

Our results also showed that high socioeconomic status has a positive relationship to global QOL and utility. A high socioeconomic status was associated with a significantly increased survival time [14]. Additionally, Kawachi and Berkman illustrate several pathways that can affect psychological well-being through participation in social networks [32]. Moreover, social network correlated positively with social support (0.51) and was positively regressed on income (beta = 0.002) [15]. Furthermore, strong correlations were noted between HRQOL and health and disability scores (− 0.58) [15]. Herein, we also found a significant relationship for economic status with global QOL and utility. As mentioned previously, social welfare systems may need to offer more support to HNC patients with a lower annual family income to increase the QOL and utility.

QLQ-H&N35 can detect a significant deterioration of symptoms after treatment in HNC patients [33]. Previous investigations showed cutoff scores on the EORTC QLQ-C30 and QLQ-H&N35 of 90 on physical functioning, role functioning, and emotional functioning; 80 on global quality of life; and 20 on fatigue. These cutoff scores may be helpful for HNC patients who require more attention [34, 35]. In the current study, total patient results with respect to physical functioning, role functioning, and social functioning match the previously defined cutoff scores. The emotional function scores of oropharyngeal, hypopharyngeal and laryngeal cancer patients in the current study were similar to the cutoff scores reported by Jansen and colleagues [34]. Hammerlid et al. [3] suggested that the fatigue of HNC patients in the Swedish and Dutch populations was similar and could be predicted by the EORTC QLQ-C30. However, the fatigue domain in the current study did not fit the previous reports, except for the laryngeal group. The possible reasons may include race, proportion of head and neck cancer (40% oral cavity cancer in our study and 26% in the Dutch population), and different treatment types (greater than 60% of HNC patients received chemotherapy or radiotherapy in the current study; however, only 20–25% of HNC patients received chemotherapy or radiotherapy in the Dutch study) [34].

Compared with hypopharyngeal/laryngeal cancer, patients with oral/oropharyngeal cancer reported more oral pain and sexual problems but fewer speech problems [36]. Similarly, oral/oropharyngeal cancer patients reported more oral pain than hypopharyngeal/laryngeal cancer in the current study. Additionally, more speech problems were found in laryngeal cancer patients than oral cancer patients. Interestingly, in our study, sexual problems were more often noted in patients with oropharyngeal cancer than other cancer subsets. Moreover, this group of patients had more problems about swallowing, appetite loss, dental problems, dry mouth, sticky saliva and malaise. Malnutrition and reduced ability to swallow along with loss of appetite were correlated to 2-year survival in a previous study [37]. Therefore, the potential risk of malnutrition in oropharyngeal cancer patients should be given attention for clinical treatment.

There are some limitations in this study. First, those earning less than 500,000 NTD have a lower quality of life and utility score in the current study. However, the sample size of patients was limited, making statistical conclusions very tentative. Second, it was not easy to assess utility in head and neck cancer patients using the TTO method. However, Noel et al. [25] demonstrate that TTO could generate significantly higher mean HU scores than the EQ-5D and the HUI3 in a population with head and neck cancer. Hamilton DW et al. [7] used the TTO to assess the factors influencing patients’ decisions in advanced laryngeal cancer. Although the EQ-5D and HUI3 seemed be more capable of discriminating utility differences between subsets of patients with head and neck cancer and correlate well with each other when compared with TTO [25]. In the current study, twenty-five (20%) patients were unwilling to exchange years of life for better health. According to Ringash et al., 24% of 120 laryngeal cancer patients could not assess utility. Ottoet al. attempted to evaluate the impact of laryngectomy in 46 post-laryngectomy patients and found that 80.4% of the patients would not be willing to trade off expectancy of QOL for voice preservation. Other methods, such as EQ-5D use to assess utility scores, may offer more information on this issue. New empirical evidence suggests that aside from sociodemographic and clinical parameters, tumor- and patient-related biomarkers and psychological functioning may also be related to the course of QOL and survival in cancer patients [38, 39]. Given that the current study is cross-sectional, future longitudinal follow-up would be beneficial and can provide more information. Additionally, large-scaled cohort studies are needed to investigate the association between RQOL and survival in HNC patients in relation to broadly defined possible moderating factors, including cancer-related, personal, genetic, biological, psychological, physical, lifestyle-related, and social determinants [38, 39]. Otherwise, the findings of quality of life and utility may not be comparable although people in both Swedish and Taiwan have a relatively high-quality health care system.

## Conclusion

Head and neck cancer disease and treatment lead to deterioration. Compared with other tumor locations, patients with oropharyngeal cancer may have more problems to address, such as malnutrition. The disability will lead to poor health-related QOL and utility. Economic status may contribute to health-related QOL and utility, while marital status is related to utility for head and neck cancer patients.

## Abbreviations

3DCRT: Three-dimensional conformal radiation therapy
EORTC QLQ-C30: European Organization for Research and Treatment of Cancer Quality of life Questionnaire-Core 30
EQ-5D: EuroQoL instrument
HNC: Head and neck cancer
HRQOL: Health-related quality of life
HSUV: Health state utility value
HT: Helical tomotherapy
HUI3: HU Index Mark 3
IMRT: Intensity modulated radiation therapy
QOL: Quality of life
SDs: Standard deviations
TTO: Time trade off
VMAT: Volumetric modulated arc therapy

## References

[1] Pulte D, Brenner H (2010). Changes in survival in head and neck cancers in the late 20th and early 21st century: a period analysis. Oncologist.

[2] Funk GF, Karnell LH, Christensen AJ (2012). Long-term health-related quality of life in survivors of head and neck cancer. Arch Otolaryngol Head Neck Surg.

[3] Hammerlid E, Adnan A, Silander E (2017). Population-based reference values for the European Organization for Research and Treatment of Cancer head and neck module. Head Neck.

[4] Pais-Ribeiro JL (2004). Quality of life is a primary end-point in clinical settings. Clin Nutr.

[5] Group W. The World Health Organization Quality of Life assessment (WHOQOL): position paper from the World Health Organization. Soc Sci Med. 1995;41(10):1403–9. 10.1016/0277-9536(95)00112-k 8560308

[6] Weiss MH, Harrison LB, Isaacs RS (1994). Use of decision analysis in planning a management strategy for the stage N0 neck. Arch Otolaryngol Head Neck Surg.

[7] Hamilton DW, Bins JE, McMeekin P, Pedersen A, Steen N, De Soyza A, Thomson R, Paleri V, Wilson JA (2016). Quality compared to quantity of life in laryngeal cancer: a time trade-off study. Head Neck.

[8] Laccourreye O, Malinvaud D, Menard M, Consoli S, Giraud P, Bonfils P (2014). Total laryngectomy or laryngeal preservation for advanced laryngeal cancer. Impact of the functional risk upon the patient’s preferences. Eur Ann Otorhinolaryngol Head Neck Dis.

[9] Hammerlid E, Taft C (2001). Health-related quality of life in long-term head and neck cancer survivors: a comparison with general population norms. Br J Cancer.

[10] Nordgren M, Hammerlid E, Bjordal K, Ahlner-Elmqvist M, Boysen M, Jannert M (2008). Quality of life in oral carcinoma: a 5-year prospective study. Head Neck.

[11] Ringash J, Redelmeier DA, O'Sullivan B, Bezjak A (2000). Quality of life and utility in irradiated laryngeal cancer patients. Int J Radiat Oncol Biol Phys.

[12] Lillard LA, Panis CW (1996). Marital status and mortality: the role of health. Demography.

[13] Umberson D (1987). Family status and health behaviors: social control as a dimension of social integration. J Health Soc Behav.

[14] Du XL, Lin CC, Johnson NJ, Altekruse S (2011). Effects of individual-level socioeconomic factors on racial disparities in cancer treatment and survival: findings from the National Longitudinal Mortality Study, 1979-2003. Cancer.

[15] Tobiasz-Adamczyk B, Galas A, Zawisza K, Chatterji S, Haro JM, Ayuso-Mateos JL, Koskinen S, Leonardi M (2017). Gender-related differences in the multi-pathway effect of social determinants on quality of life in older age-the COURAGE in Europe project. Qual Life Res.

[16] Chie WC, Hong RL, Lai CC, Ting LL, Hsu MM (2003). Quality of life in patients of nasopharyngeal carcinoma: validation of the Taiwan Chinese version of the EORTC QLQ-C30 and the EORTC QLQ-H&N35. Qual Life Res.

[17] Chie WC, Yang CH, Hsu C, Yang PC (2004). Quality of life of lung cancer patients: validation of the Taiwan Chinese version of the EORTC QLQ-C30 and QLQ-LC13. Qual Life Res.

[18] Bjordal K, Hammerlid E, Ahlner-Elmqvist M, de Graeff A, Boysen M, Evensen JF, Biorklund A, de Leeuw JR, Fayers PM, Jannert M (1999). Quality of life in head and neck cancer patients: validation of the European Organization for Research and Treatment of Cancer quality of life questionnaire-H&N35. J Clin Oncol.

[19] Aaronson NK, Ahmedzai S, Bergman B, Bullinger M, Cull A, Duez NJ, Filiberti A, Flechtner H, Fleishman SB, de Haes JC (1993). The European Organization for Research and Treatment of Cancer QLQ-C30: a quality-of-life instrument for use in international clinical trials in oncology. J Natl Cancer Inst.

[20] Fayers PM, Aaronson NK, Bjordal K, Gronvold M, Curran D, Bottomley A (2001). EORTC QLQ-C30 Scoring Manual.

[21] Whitehead SJ, Ali S (2010). Health outcomes in economic evaluation: the QALY and utilities. Br Med Bull.

[22] O'Brien BJ (1997). Health state utility anchors: being clear on what “1” means.

[23] Weiss M (1997). Use of decision analysis in planning a management strategy for the stage N0 neck. Arch Otolaryngol Head NeckSurg.

[24] Hammerlid E, Silander E, Hornestam L, Sullivan M (2001). Health-related quality of life three years after diagnosis of head and neck cancer--a longitudinal study. Head Neck.

[25] Noel CW, Lee DJ, Kong Q, Xu W, Simpson C, Brown D, Gilbert RW, Gullane PJ, Irish JC, Huang SH (2015). Comparison of health state utility measures in patients with head and neck Cancer. JAMA Otolaryngol Head Neck Surg.

[26] Del Barco Morillo E, Mesia R, Adansa Klain JC, Vazquez Fernandez S, Martinez-Galan J, Pastor Borgonon M, Gonzalez-Rivas C, Caballero Daroqui J, Berrocal A, Martinez-Trufero J (2016). Phase II study of panitumumab and paclitaxel as first-line treatment in recurrent or metastatic head and neck cancer. TTCC-2009-03/VECTITAX study. Oral Oncol.

[27] Loimu V, Makitie AA, Back LJ, Sintonen H, Rasanen P, Roine R, Saarilahti K (2015). Health-related quality of life of head and neck cancer patients with successful oncological treatment. Eur Arch Otorhinolaryngol.

[28] Govers TM, Schreuder WH, Klop WM, Grutters JP, Rovers MM, Merkx MA, Takes RP (2016). Quality of life after different procedures for regional control in oral cancer patients: cross-sectional survey. Clin Otolaryngol.

[29] Woodard TD, Oplatek A, Petruzzelli GJ (2007). Life after total laryngectomy: a measure of long-term survival, function, and quality of life. Arch Otolaryngol Head Neck Surg.

[30] Aizer AA, Chen MH, McCarthy EP, Mendu ML, Koo S, Wilhite TJ, Graham PL, Choueiri TK, Hoffman KE, Martin NE (2013). Marital status and survival in patients with cancer. J Clin Oncol.

[31] Pearlin LI, Johnson JS (1977). Marital status, life-strains and depression. Am Sociol Rev.

[32] Kawachi I, Berkman LF (2001). Social ties and mental health. J Urban Health.

[33] Bjordal K, de Graeff A, Fayers PM, Hammerlid E, van Pottelsberghe C, Curran D, Ahlner-Elmqvist M, Maher EJ, Meyza JW, Bredart A (2000). A 12 country field study of the EORTC QLQ-C30 (version 3.0) and the head and neck cancer specific module (EORTC QLQ-H&N35) in head and neck patients. EORTC Quality of Life Group. Eur J Cancer.

[34] Jansen F, Snyder CF, Leemans CR, Verdonck-de Leeuw IM (2016). Identifying cutoff scores for the EORTC QLQ-C30 and the head and neck cancer-specific module EORTC QLQ-H&N35 representing unmet supportive care needs in patients with head and neck cancer. Head Neck.

[35] Snyder CF, Blackford AL, Sussman J, Bainbridge D, Howell D, Seow HY, Carducci MA, Wu AW (2015). Identifying changes in scores on the EORTC-QLQ-C30 representing a change in patients’ supportive care needs. Qual Life Res.

[36] Verdonck-de Leeuw IM, Buffart LM, Heymans MW, Rietveld DH, Doornaert P, de Bree R, Buter J, Aaronson NK, Slotman BJ, Leemans CR (2014). The course of health-related quality of life in head and neck cancer patients treated with chemoradiation: a prospective cohort study. Radiother Oncol.

[37] Hammerlid E, Wirblad B, Sandin C, Mercke C, Edstrom S, Kaasa S, Sullivan M, Westin T (1998). Malnutrition and food intake in relation to quality of life in head and neck cancer patients. Head Neck.

[38] Barsevick A, Frost M, Zwinderman A, Hall P, Halyard M, Consortium G (2010). I’m so tired: biological and genetic mechanisms of cancer-related fatigue. Qual Life Res.

[39] Lutgendorf SK, Sood AK, Antoni MH (2010). Host factors and cancer progression: biobehavioral signaling pathways and interventions. J Clin Oncol.

